# A review of African horse sickness and its implications for Ireland

**DOI:** 10.1186/2046-0481-65-9

**Published:** 2012-07-05

**Authors:** Geoffrey M Thompson, Stephen Jess, Archie K Murchie

**Affiliations:** 1 School of Biological Sciences, Queen’s University of Belfast, Belfast, BT7 1NN, Northern Ireland; 2 Agri-Food and Biosciences Institute, Newforge Lane, Belfast, BT9 5PX, Northern Ireland

**Keywords:** African horse sickness, *Culicoides* spp., Epizootic disease, Ireland

## Abstract

African horse sickness is an economically highly important non-contagious but infectious Orbivirus disease that is transmitted by various species of *Culicoides* midges. The equids most severely affected by the virus are horses, ponies, and European donkeys; mules are somewhat less susceptible, and African donkeys and zebra are refractory to the devastating consequences of infection. In recent years, Bluetongue virus, an Orbivirus similar to African horse sickness, which also utilises *Culicoides* spp. as its vector, has drastically increased its range into previously unaffected regions in northern Europe, utilising indigenous vector species, and causing widespread economic damage to the agricultural sector. Considering these events, the current review outlines the history of African horse sickness, including information concerning virus structure, transmission, viraemia, overwintering ability, and the potential implications that an outbreak would have for Ireland. While the current risk for the introduction of African horse sickness to Ireland is considered at worst ‘very low’, it is important to note that prior to the 2006 outbreak of Bluetongue in northern Europe, both diseases were considered to be of equal risk to the United Kingdom (‘medium-risk’). It is therefore likely that any outbreak of this disease would have serious socio-economic consequences for Ireland due to the high density of vulnerable equids and the prevalence of *Culicoides* species, potentially capable of vectoring the virus.

## Introduction

African horse sickness (AHS) is a disease caused by the African horse sickness virus (AHSV). The disease affects horses, ponies, and European donkeys most severely; mules are somewhat less affected, and African donkeys and zebra are refractory to the devastating consequences of infection [1-4]. The virus is a non-contagious, vector-borne Orbivirus that is transmitted primarily by female *Culicoides* midges during a blood meal, which they require for reproduction [4]. In addition to equids, camels, goats, and buffalo can become infected [5]. Additionally, some carnivores such as dogs, can become infected via ingestion of contaminated meat. However, there have been no documented cases of transmission of AHSV in carnivores in the wild, and it is considered that they are a ‘dead-end’ host, rather than a reservoir of infection [6,7]. Owing to the potential of this virus to cause widespread death and debilitating disease in naïve equid populations, it is listed as a notifiable equine disease by the World Organisation for Animal Health (OIE), which makes outbreaks of the disease compulsorily notifiable to the OIE. Such occurrences can result in serious consequences for international trade of animals and animal products for the affected country [8]. It is currently predicted, that a widespread outbreak of this disease would have a devastating effect on the horse industry of any country affected [9].

## History

The first recorded reference of AHS occurred in 1327 in Yemen [10], but it is most likely that the virus originated on the African continent where it could have been transmitted in the natural zebra population [2]. The disease was first recognised around 60 years after the initial introduction of horses to Africa in 1657, and the first major outbreak occurred in 1719. This outbreak resulted in the deaths of approximately 1, 700 animals [11]. Subsequently, there has been at least an additional 10 major (and several lesser) outbreaks of AHS on the African continent [2]. These outbreaks typically coincide with warm-phase El niňo southern oscillation (ENSO) events [12] and they have had devastating results for the African horse population, most notably, the 1854–1855 outbreak in South Africa, which resulted in the deaths of around 70, 000 animals [13,14]. Throughout the last century, there has been a steady decline in the number of cases of AHS in South Africa, which correlated with a decline in the number of wild zebra due to hunting. Currently, AHS is not considered to be endemic in the greater part of South Africa, with the exception of the North-eastern Lowveld of Mpumalanga Province where seroconversion of AHSV occurs in zebra in every month of the year and virtually all adult animals have specific antibodies to all nine serotypes of the virus. Typically, the disease first appears in the North-eastern part of the country in December/January and subsequently spreads southwards, occasionally reaching as far as the Western Cape Province [14]. However, it has been reported that restocking of zebra is occurring in many areas of South Africa and that at some point, conditions for the re-establishment for AHS in other regions of the country may occur [2]. These conditions appear to have been reached in recent years, with a number of outbreaks of AHSV serotype 1 (AHSV-1) recorded in the Western Cape Province, with a horse case fatality rate of more than 90% [15].

While AHS is considered to be primarily an African disease, there have been a number of epizootic events that have occurred outside this region. The most severe of which occurred in Asia between 1959–1961, when AHSV serotype 9 (AHSV-9) spread throughout Afghanistan, Cyprus, India, Iraq, Jordan, Lebanon, Pakistan, Saudi Arabia, and Syria resulting in the deaths of around 300, 000 equids [2,3,16-18]. This outbreak was followed by another epizootic of AHSV-9 in 1965 which spread throughout Morocco, Algeria, Tunisia and Spain. It is believed that the movement of nomads and their donkeys across the Sahara was responsible the appearance of AHSV-9 in northern Africa [2].

Following these outbreaks, AHS was confined to sub-Saharan Africa for around 20 years until July 1987, when AHSV serotype 4 (AHSV-4) was reported in central Spain [19]. This serotype had never before been recorded outside of Africa [20], and it is believed that the outbreak was caused by the importation of a number of sub-clinically infected zebra from Namibia to a safari park at Aldea del Fresno [19]. This site subsequently became the foci for the first 27 cases of AHS in Europe [21]. Over the following months, the outbreaks continued to spread within central Spain until October, when temperatures became too low for the virus transmission cycle to continue. It was initially hoped that this break in the transmission cycle would remove the infection from the region, but this was not the case, and AHSV successfully overwintered in the European climate. Subsequently, new outbreaks occurred in Spain (1988–1990), Portugal (1989) and Morocco (1989–1991) [20] indicating that the virus had persisted in the area for at least five years and successfully overwintered on four occasions. In total, the outbreak in Spain resulted in the deaths of over 400 equids, and a further 900 were destroyed in attempts to control the virus spread. It was not until 1990, when a monovalent vaccine was administered to more than 350, 000 susceptible animals, that the outbreak was controlled [20,22]. Portugal reported a total of 206 cases, but here the infection was removed quickly following a mass vaccination policy, eradication on infected farms, and strict animal movement controls. The total cost of the 13-week incursion into Portugal was estimated at around US $ 2 million [23]. While this figure may seem high compared to the total number of deaths (US $ 9, 493/mortality), when compared to the Portuguese equine population as a whole, the total cost of this campaign appears relatively small (170, 000 animals, US $ 11.50/animal). This campaign must therefore be seen as successful, as it drastically reduced the potential damage to the Portuguese equine industry [23].

## The African horse sickness virus

There are currently nine recognised serotypes of AHSV worldwide, the last of which was identified in 1960 [24]. The AHSV is a double-stranded RNA virus from the genus Orbivirus (family Reoviridae), and as such, it is morphologically similar to other Orbiviruses such as Bluetongue virus (BTV), Epizootic haemorrhagic disease virus (EHDV), and Equine encephalosis virus (EEV) [1,7,25,26]. The virion, which is approximately 70 nm in diameter, is made up of a two-layered icosahedral capsid [13]. This capsid encloses the genome which comprises ten segments of linear double stranded RNA [26,27]. The outer shell of the capsid comprises two structural proteins (VP2 and VP5), which are involved in virus attachment and cell entry. These are the most variable proteins and as such are used to determine the virus serotype (usually VP2) [4,28-31].

## Pathogenesis

Despite the distinct differences in the clinical severity of AHS infection in different equids, the typical pattern of pathogenesis is similar. Following exposure through the bite of an infected vector, the virus initially replicates in an adjacent lymph node before disseminating throughout the entire body via the circulatory system. This is the primary viraemia which leads to infection of the lungs and lymphoid tissues. The virus then undergoes a further replication cycle in these target organs which in turn gives rise to a secondary viraemia. The virus has a predilection for the vascular endothelial cells throughout the body, causing extensive damage including effusions into body cavities and tissues, and widespread haemorrhages [13]. The duration of this process from initial infection to secondary viraemia can vary between 2–21 days, although it usually takes less than nine [32]. In horses, the period of viraemia usually lasts between 4–8 days, whereas in donkeys, it may persist for up to four weeks. In zebra, this period may be extended to approximately 40 days post-infection [4,13,18,33]. In common with BTV, AHSV is often associated with red blood cells of the infected animal throughout both primary and secondary viraemia. In ruminants infected with BTV, this often leads to an extended period of viraemia, although this does not appear to be the case with AHS [2].

## Clinical signs

AHSV displays itself in the form of four diseases: cardiac form, pulmonary form, mixed form, and horse sickness fever [34].

**Figure 1:**
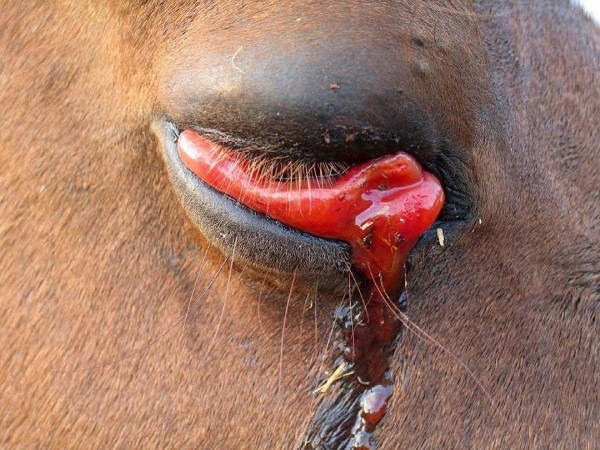
**Horse suffering from petechial haemorrhage.** Picture courtesy of the Institute for Animal Health, Pirbright.

The cardiac form of the disease is usually characterised by the development of a fever, oedema of the head, neck, chest, supraorbital fossae, petechial haemorrhages in the eyes (Figure 1), ecchymotic haemorrhages on the tongue, and colic. In these cases, mortality of infected animals may exceed 50% [13].

**Figure 2:**
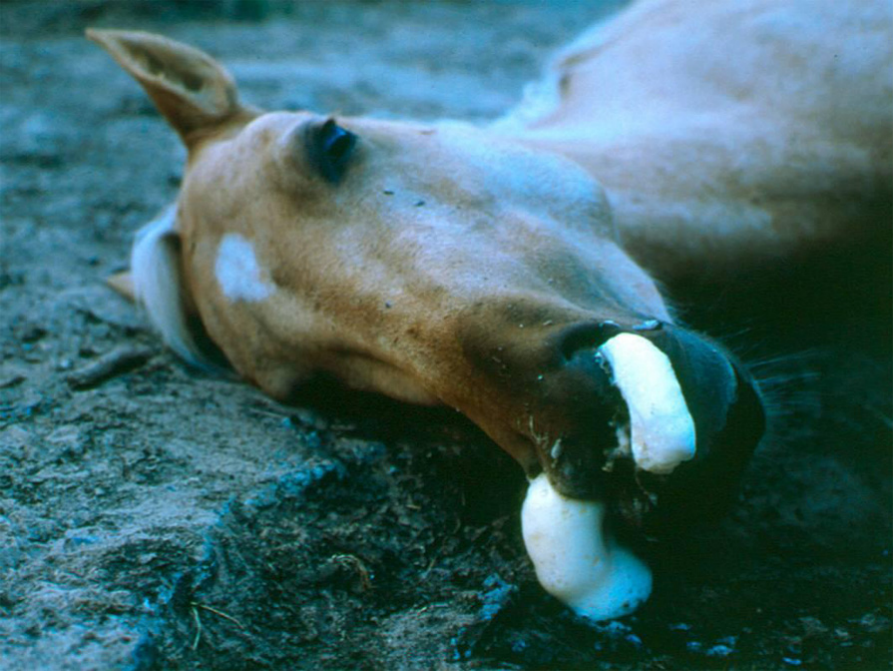
**Horse suffering from severe respiratory distress.** Picture courtesy of the Institute for Animal Health, Pirbright.

The pulmonary form of the disease is the most serious. It is typically associated with a rapid onset of symptoms which include the development of a fever, depression, severe respiratory distress (Figure 2), severe dyspnoea, coughing, and sweating. Typically, the mortality rates of this form of the disease exceed 95% [13].

The mixed form is often the most common form of the disease and as the name suggests, it is a combination of the cardiac and the pulmonary form. Typically, the mortality rate may exceed 70%, and death can often occur within three to six days [13].

**Figure 3:**
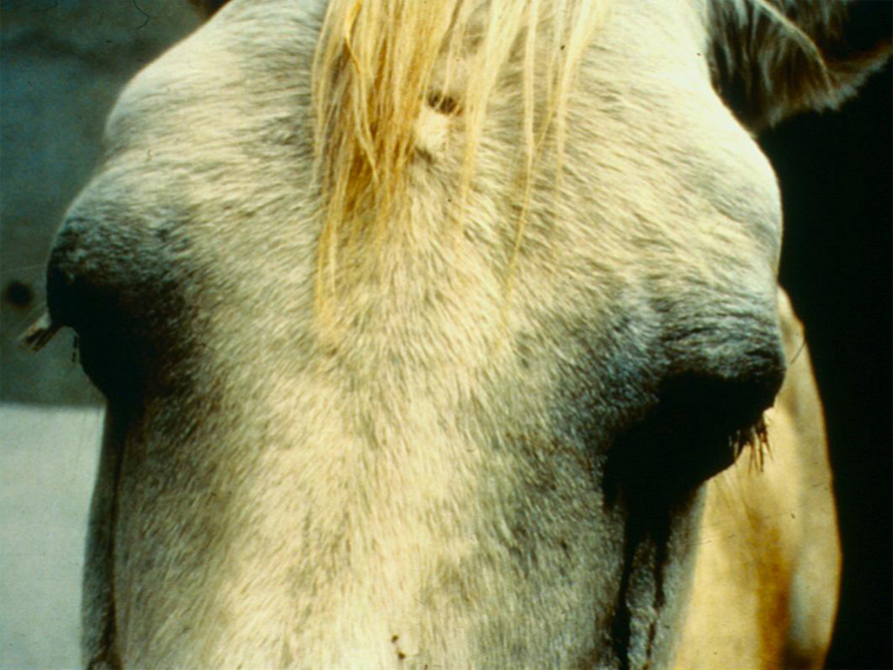
**Horse exhibiting oedema of the supraorbital fossae.** Picture courtesy of the Institute for Animal Health, Pirbright.

Horse sickness fever is the mildest form of the disease, during which, the animal will develop a moderate fever and some oedema of the supraorbital fossae (Figure 3). There is no mortality associated with this form of the disease [13].

## The African horse sickness vector

The principal vector of AHSV is the female midge of the genus *Culicoides* (Diptera: Ceratopogonidae). These insects are important vectors of a number of viral diseases worldwide including: Akabane, Bovine ephemeral fever, Bluetongue, Epizootic haemorrhagic disease, Equine encephalosis, Oropouche, and The Palyams [1,35]. It was first discovered that adult females from the genus *Culicoides* were the principal vector for AHSV in 1944 [36]. Today there are approximately 1, 340 recognised species of *Culicoides* worldwide, of which 96% are obligate bloodsuckers [37]. Typically, *Culicoides* spp. measure between 1–3 mm, making them amongst the smallest haematophagous flies in the world, and they are currently found on all continents (except Antarctica) from sea level up to around 4, 200 m (in Tibet) [38].

The principal vector of AHSV worldwide is *Culicoides imicola*[39], which is a common species distributed throughout Africa, much of Southeast Asia, and southern Europe [2]. It has been reported that over 1 million individuals may be captured in a single light trap per night [40]. It was previously considered that *C. imicola* was the only species of *Culicoides* capable of vectoring AHSV, but other species have been identified as potential vectors. A laboratory study in 1975 found that the American BTV vector, *Culicoides variipennis* (*sonorensis*), also has the ability to transmit AHSV [41]. Additionally, AHSV has also been isolated from the northern European BTV vectors, *Culicoides obsoletus* and *Culicoides pulicaris*, during the AHSV-4 outbreak in Spain [42,43]. However, it is important to note that the presence of virus does not necessarily indicate that these midges are capable of virus transmission. More recently, *Culicoides bolitinos* has been implicated as a vector in Africa, particularly at higher altitudes where *C. imicola* is rare [44,45].

While *Culicoides* spp. are widely considered to be the principal vector for the transmission of AHSV, a number of other species have been implicated in its transmission. Laboratory experiments have shown that some species of mosquito, most notably *Aedes aegyptiAnopheles stephensi*, and *Culex pipiens*, are capable of AHSV infection and transmission [4,41,46-49]. Although, the virus only underwent multiplication in a limited number of mosquitoes and the maximum virus titre was not appreciably higher than the amounts ingested, an eclipse phase occurred subsequent to infection and virus was recovered for at least five weeks [41]. Additionally, AHSV has also been isolated in the field from samples of camel ticks, *Hyalomma dromadarii*, in Egypt [50]. However, the prevailing scientific opinion suggests that the role of these species in the transmission of AHSV is likely to be insignificant [4].

## Overwintering

**Figure 4:**
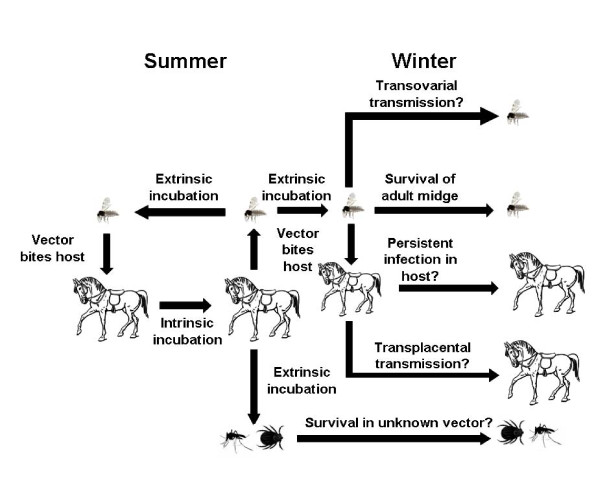
**The normal transmission cycle of African horse sickness virus in summer (Left) and possible overwintering mechanisms (Right).** Image created using Microsoft Clip Art with permission from Microsoft PowerPoint (2007).

Throughout the majority of the geographic range of AHSV, the climate is suitable for vector activity and viral replication to occur throughout the year [4]. For example, in northeast South Africa it has been found that seroconversion of AHSV occurs in every month of the year in zebra [2]. However, in some regions, conditions are not suitable for year round vector activity or viral replication, and a break in the transmission cycle occurs e.g. Spain (1987–1990). This poses the question as to where and how does the virus survive over the winter period? Similar to BTV, there are a number of possible mechanisms by which AHSV could be overwintering (Figure 4): 1) in the vector population, 2) in the host population, 3) via an alternative transmission cycle involving an unknown vector or host [4].

### 1) Survival in the vector

There are two possible means by which AHSV may survive the winter in the adult vector population. The first is via transovarial transmission (vertical transmission of AHSV from adult females to their eggs). While there is evidence that vertical transmission of arboviruses occurs in mosquitoes and sandflies [35,51,52], to date, no evidence of live AHSV has been recovered in any larval *Culicoides* spp.. Hitherto, only fragments of BTV RNA have been found in larvae and further research is required in order to fully understand if this route of transmission exists [53].

A second, better understood method, involves virus retention in adult *Culicoides* which survive the winter drop in temperatures. *Culicoides imicola* activity has been recorded at temperatures as low as 12°C [54,55]. At these temperatures, the life span of some *Culicoides* spp. can be extend up to 90 days [55,56]. While viral replication ceases at temperatures below 15°C, once temperatures raise above this point, viral replication may resume in a number of individuals [55]. Under these circumstances, AHSV may overwinter within the adult vector population, provided some infected individuals survive through the winter period. This mechanism was implicated in the successful overwintering of AHSV in Spain, Portugal, and Morocco [39,57].

### 2) Survival in the vertebrate host

While the survival of AHSV in adult *Culicoides* spp. remains a better understood mechanism for virus overwintering, there are a number of possible means by which the virus may overwinter within the vertebrate host. Whilst persistent or chronic infection in horses is considered unlikely as the period of viraemia is short (4–8 days) and the mortality rates are high (often > 95%) [13], donkeys may remain viraemic for up to four weeks, and the viraemic period of zebra can extend up to around 40 days [4,13,18,33]. This creates the possibility that chronically infected animals may represent a possible mechanism for overwintering of AHSV, although there is no current field evidence to support this hypothesis [4].

Another recently discovered overwintering mechanism for Orbiviruses is the possibility of vertical transmission within the host. To date, there has been no evidence to suggest that this route occurs for AHSV, but recent studies involving BTV-8 in northern Europe have demonstrated that this mechanism occurs within cattle [58], and it may be possible that it also occurs for AHSV, although further research is required to establish if this mechanism exists.

### 3) Survival in an unknown vector or host

The final mechanism by which AHSV may be overwintering, is in a vector species other than *Culicoides* such as ticks, which can live for several years, easily bridging the winter gap [59]. It has long been known that AHSV has been isolated in the field from samples of camel ticks, *Hyalomma dromadarii*[50], and a number of experiments have shown that some species of mosquito are capable of AHSV infection and transmission [4,46-49]. However, the prevailing scientific opinion suggests that the role of these species in the overwintering of AHSV is likely to be negligible [4], and further research is required to evaluate what role, if any these species play.

## Implications for Ireland

The Republic of Ireland (R.O.I.) has a longstanding equine tradition, and it is therefore unsurprising that it has the highest density of sport horses in Europe [60]. A recent estimate valuated the industry in excess of € 1.1 billion per annum [61]. The current national equid population is estimated at approximately 110, 000 animals and it is believed that 53, 000 people are regularly involved in horse riding activities [60]. A conservative figure of the annual expenditure within the industry is estimated at approximately € 400 million [60], while the value of various racing festivals and other meetings to the local economy was valued at € 260 million [61]. Similarly, in Northern Ireland (N.I.), it is estimated that around £ 110 million is spent per annum on equine services and products within the economy, with a further £ 2 million expended outside of N.I. on thoroughbred stud fees. In total, the equid population is estimated at approximately 36, 000 animals with a value of around £ 108 million. The sector uses approximately 30, 000 acres of land (> 1% of total agricultural land in N.I.), and is an important employer within N.I. with the core equine industry supporting 5, 657 full-time equivalents, equating to a labour value of around £ 54 million. In addition, the capital value of buildings and major equipment is estimated at approximately £ 121 million, with the two race courses at Down Royal and Downpatrick generating approximately £ 1.7 million per annum in sales revenue [62].

Whilst any incursion of AHSV into Ireland is likely to be relatively short lived due to the absence of any suitable reservoir host, even an isolated case, where infection is successfully contained, could result in serious economic consequences due to long-term restrictions on trade. Furthermore, in the event of a widespread outbreak, the direct loss of horses combined with the introduction of movement restrictions, could have a potentially devastating effect on the industry. It is estimated that over half the economic impact of the sector could be lost within one to two years, with irreparable damage caused to the horse racing sector and other sporting disciplines [9].

There are three main pathways by which AHS could be introduced to Ireland: importation of infected equidae, introduction of infected vectors, and importation of infected biological substances/germplasm [63]. Of the 843 horses imported into R.O.I. in 2009 and 2010, only four originated from a country where AHS had been reported (South Africa) [63]. Due to the strict controls on the export of horses and their products from this region, a qualitative risk assessment predicted that the risk of introduce AHSV via this route was ‘very low’[63]. The same report predicted that the risk of introduction of AHSV via wind dispersal of infected vectors was currently ‘negligible’ due to the fact that AHSV has not been recognised in any country neighbouring Ireland. However, previous evidence has shown that *Culicoides* can be transported up to 700 km via wind dispersal and this was the most likely transmission route for the introduction of AHSV into Cyprus (1960), Spain (1966), and the Cape Verde Islands (1943 & 1999) [64,65]. Furthermore, this method is seen as the most likely route for the introduction of Bluetongue and more recently, Schmallenberg virus into the U.K. from mainland Europe [66,67]. This ‘negligible’ status should therefore be revised in the event of any outbreak being reported in a country of close proximity. Additionally, the report considered the risk of AHSV introduction via the accidental importation of infected *Culicoides* by ‘hitch-hiking’ on trucks transporting livestock or plants to be ‘negligible’ as there is no evidence in the literature to suggest that such accidental transportation has ever occurred. Finally, the report considered that the risk of AHSV importation in germplasm/biological substances was ‘negligible’, as hitherto there has been no recorded outbreak of AHS due to the use of infected semen, ova, or embryos [63]. Overall, the current predicted risk of AHSV to the U.K. and Ireland is considered ‘negligible’ to ‘very low’ [63,68]. Nonetheless, it is important to note that these reports did not consider the introduction of AHSV via the illegal importation of infected equidae and/or their products. Furthermore, the predicted risk of an incursion of AHSV or BTV into the U.K. prior to the 2006 BT outbreak was considered to be of ‘Medium-risk’[69]. However, outbreaks of BTV have subsequently been reported in the U.K. in 2007 [70] and in N.I. in 2008 [58]. For these reasons, and in consideration of the recent unexplained outbreaks of BTV-8 in northern Europe [71], it is important that Ireland is suitably prepared should AHSV be imported into the island.

In response to the outbreak of BTV in northern Europe, the BTV vector surveillance programme was initiated in Ireland [72]. This programme examined the activity of *Culicoides* spp. for one night per week at 34 farmland trapping sites throughout the year. In total, approximately half a million midges, from 21 species of *Culicoides* were identified*.* The maximum number of *Culicoides* caught in one night was in excess of 21,000 midges in the R.O.I. [72]. A similar surveillance network in N.I. operating at 14 trapping sites caught a maximum of around 36,000 *Culicoides* per night in N.I. [Dr. Stephen Jess, unpublished observations]. Of the *Culicoides* identified, approximately 73% were considered to be potential AHSV vectors (Obsoletus group, 50.6%; Pulicaris group 22%) [72]. Initial results indicate that the vector period appears to operate between the beginning of April until mid-November/early December, although midge abundance correlates with weather conditions, most notably wind and temperature [72]. Therefore, it is evident that if AHSV were to be introduced into Ireland there are sufficient vectors present to enable the spread of the virus.

## Conclusions

It is evident from previous outbreaks of AHSV worldwide that this disease can have serious consequences for animal health and mortality. As such, it is likely that any introduction to Ireland would be catastrophic, considering the high population density of vulnerable animals, and the importance of the equine industry to the rural economy. Despite the current risk of importation of AHSV being considered at worst ‘very low’, a stockpile of live, attenuated vaccines, which are currently used in sub-Saharan Africa, has been created for the potential use in the European Union should an outbreak occur [9]. These vaccines are not currently considered to be an option for Ireland, as there may be a risk of the vaccine reverting to the wild-type virus, causing virulence and spreading the disease [9]. However, DEFRA has recently initiated a three year project with the aim of developing a safer and more effective vaccine against AHSV [73]. Consequently, there is an important need for continued vigilance in Ireland with regard to arthropod-borne diseases such as AHSV and BTV in order to protect livestock, the agricultural industry, and the rural economy as a whole.

## Abbreviations

AHS = African horse sickness; AHSV = African horse sickness virus; AHSV-1 = African horse sickness virus serotype 1; AHSV-4 = African horse sickness virus serotype 4; AHSV-9 = African horse sickness virus serotype 9; BT = Bluetongue; BTV = Bluetongue virus; BTV-8 = Bluetongue virus serotype 8; DARD = Department of Agriculture and Rural Development Northern Ireland; DEFRA = Department for Environment, Food and Rural Affairs; EFSA = European Food Safety Authority; N.I. = Northern Ireland; OIE = The World Organisation for Animal Health; RNA = Ribonucleic acid; R.O.I. = Republic of Ireland.

## Competing interest

None of the authors of this manuscript has any financial or personal relationship with other people or organisations that could inappropriately influence or bias the content of this paper.

## Authors’ contributions

GMT, SJ, and AKM drafted the manuscript and compiled the literature. All authors made substantial inputs to the review, critically discussed, and approved the final manuscript.
